# Galangin Triggers Eryptosis and Hemolysis Through Ca^2+^ Nucleation and Metabolic Collapse Mediated by PKC/CK1α/COX/p38/Rac1 Signaling Axis

**DOI:** 10.3390/ijms252212267

**Published:** 2024-11-15

**Authors:** Mohammad A. Alfhili, Sumiah A. Alghareeb, Ghada A. Alotaibi, Jawaher Alsughayyir

**Affiliations:** Department of Clinical Laboratory Sciences, College of Applied Medical Sciences, King Saud University, Riyadh 12372, Saudi Arabia; 442204700@student.ksu.edu.sa (S.A.A.); galotaibi2@ksu.edu.sa (G.A.A.); jalsughayyir@ksu.edu.sa (J.A.)

**Keywords:** galangin, anticancer, eryptosis, hemolysis, calcium

## Abstract

Anticancer drugs cause anemia in patients through eryptosis and hemolysis. We thus studied the in vitro toxicity of galangin (GAL) in red blood cells (RBCs). RBCs were exposed to 50–500 μM of GAL and analyzed for markers of eryptosis and hemolysis. Ca^2+^ nucleation, phosphatidylserine (PS) externalization, oxidative stress, and cell size were detected via fluorescence-activated cell sorting using Fluo4/AM, annexin-V-FITC, 2′,7′-dichlorodihydrofluorescein diacetate, and forward scatter (FSC), respectively. Acetylcholinesterase (AChE) activity was measured via Ellman’s assay and ultrastructural morphology was examined via scanning electron microscopy. Membrane rupture and extracellular hemoglobin, aspartate transaminase (AST), and lactate dehydrogenase (LDH) were assessed via colorimetric methods. Distinct experiments were carried out to identify protective agents and signaling pathways using small-molecule inhibitors. GAL triggered sucrose-sensitive hemolysis with AST and LDH leakage, increased annexin-V-FITC and Fluo4 fluorescence, and decreased FSC and AChE activity which was associated with the formation of granulated echinocytes. Ca^2+^ omission and energy replenishment with glucose, adenine, and guanosine blunted PS externalization and preserved cellular volume. Moreover, caffeine, Trolox, heparin, and uric acid had similar ameliorative effects. Hemolysis was abrogated via caffeine, Trolox, heparin, mannitol, lactate, melatonin, and PEG 8000. Notably, co-treatment of cells with GAL and staurosporin, D4476, or acetylsalicylic acid prevented PS externalization whereas only the presence of SB203580 and NSC23766 rescued the cells from GAL-induced hemolysis. Ca^2+^ nucleation and metabolic collapse mediated by PKC/CK1α/COX/p38/Rac1 drive GAL-induced eryptosis and hemolysis. These novel findings carry ramifications for the clinical prospects of GAL in anticancer therapy.

## 1. Introduction

The anticancer potential of galangin (GAL), a flavonol extracted from lesser galangan (*Alpinia officinarum*) and honey [1], has been extensively demonstrated in previous studies. In hepatocellular carcinoma cells, GAL inhibits invasion and migration, and induces apoptosis characterized by chromatin condensation and mitochondrial dysfunction through Bax/Bcl-2/Bid/AIF/PARP/caspase signaling. GAL also downregulates H19 lncRNA and miR675 which induces p53 expression [2,3,4]. Likewise, GAL modulates ERK/JNK signaling and glutathione S-transferase P and ubiquitin carboxy-terminal hydrolase isozyme L1 to induce DNA damage in gastric cancer cells [5]. Moreover, invasion and migration of renal carcinoma cells are inhibited by GAL through E-cadherin, N-cadherin, and vimentin. GAL-induced apoptosis in these cells is associated with low superoxide dismutase, catalase, total antioxidant capacity, and lipid peroxidation [6]. GAL was also found to sensitize the cells to the cytotoxic effects of TRAIL via the Bcl-2/caspase/NF-ƙB/cFLIP/Mcl-1/survivin axis and to increase proteasome activity [7]. In breast cancer cells, GAL modulates Bax/Bcl-2/Bid/Bad/caspase signaling and inhibits PI3K/Akt to induce apoptosis. Furthermore, GAL triggers cell cycle arrest by downregulating cyclin D3, cyclin B1, CDK1, CDK2, and CDK4 and upregulating p21, p27, and p53 [8]. Similar effects were also observed in nasopharyngeal carcinoma cells in which GAL leads to cell cycle arrest and caspase-dependent apoptosis by upregulating Bak, Bax, p21, and p53, and downregulating Bcl-2, p85α, and Akt [9]. Likewise, GAL stimulates DR5 signaling in ovarian cancer cells to promote cell cycle arrest through p21 and p53, and caspase-dependent apoptosis by modulating Bax, Bcl-2, cmyc, and Akt [10]. Several modalities of cell death, including apoptosis, pyroptosis, and autophagy have also been reported in glioblastoma cells upon exposure to GAL [11]. It has also been shown that GAL triggers mitochondrial damage and apoptosis through Bax/Bcl-2/PARP and p53 signaling in bladder cancer cells [12].

Chemotherapy-related anemia is a very common complication [13] and the repertoire of anticancer drugs that cause eryptosis is growing [14]. Furthermore, the role of eryptosis is increasingly being recognized in a wide range of conditions such as diabetes mellitus, leukemia, sickle cell disease, and sepsis. Eryptotic cells release microvesicles and show decreased deformability and increased aggregability which interferes with blood flow and leads to thrombosis [15]. Comparable to apoptosis, the biochemical characteristics of eryptosis include decreased cellular volume, membrane blebbing, reduced acetylcholinesterase (AChE) activity, buildup of reactive oxygen species (ROS), and phosphatidylserine (PS) externalization. Ca^2+^ signaling is pivotal to eryptosis along with protein kinase C (PKC), p38 MAPK, casein kinase 1α (CK1α), Rac1 GTPase, and caspase [16].

Targeting eryptosis, thus, lends itself as an attractive avenue to optimize further development and validation of investigational chemotherapeutic agents. This report examined the in vitro toxic mechanisms of GAL in RBCs and informs future efforts aimed at developing GAL-based therapeutic strategies.

## 2. Results

**Figure 1:**
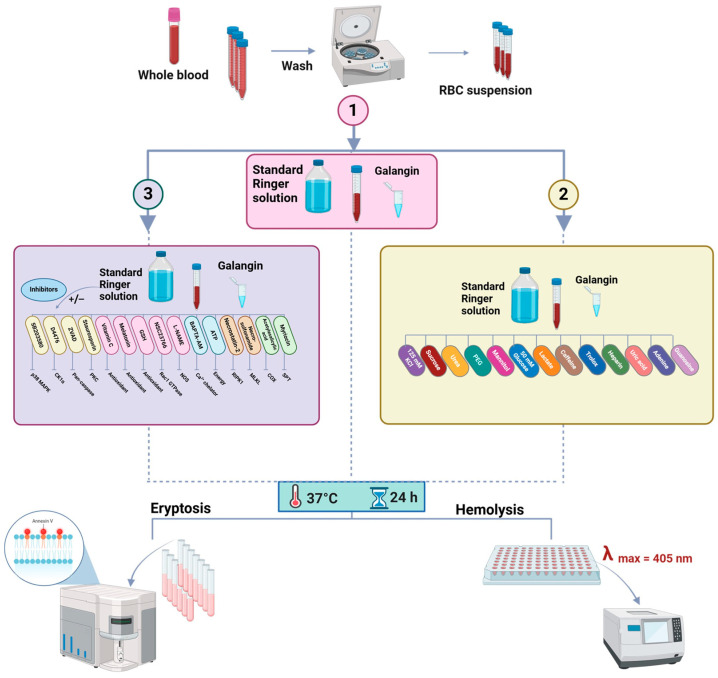
Experimental design. Prepared with BioRender.

The aim of the current study was to determine whether anticancer concentrations of GAL [3,11] exhibit eryptotic and hemolytic activities. A flowchart of the study design is shown in Figure 1.

**Figure 2:**
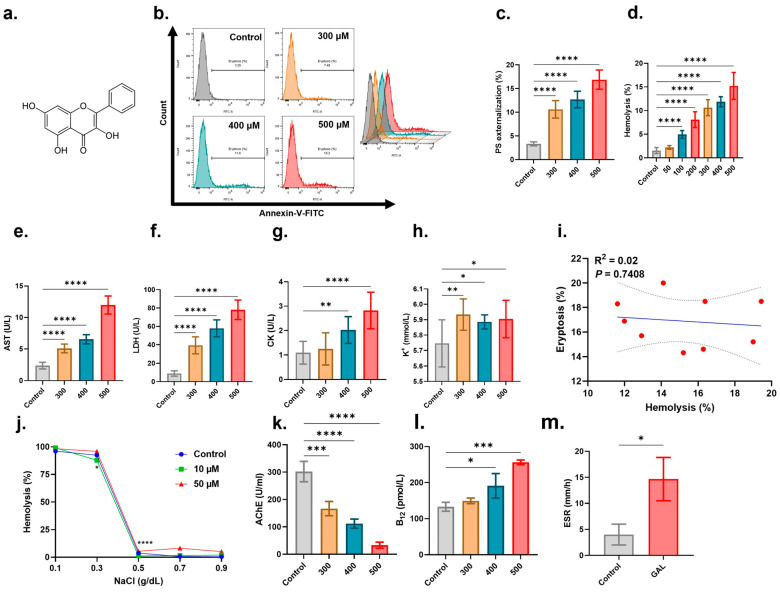
Eryptotic and hemolytic activities of GAL. (**a**) Chemical structure of GAL. (**b**) Original histograms of annexin-V-FITC fluorescence. (**c**) Percentage of eryptotic cells. (**d**) Percentage of hemolytic cells. (**e**) AST activity. (**f**) LDH activity. (**g**) CK activity. (**h**) K^+^ levels. (**i**) Correlation between eryptosis and hemolysis. (**j**) Osmotic fragility curves. (**k**) AChE activity. (**l**) B_12_ levels. (**m**) ESR. Graphs show means ± SD. * (*p* < 0.05), ** (*p* < 0.01), *** (*p* < 0.001), and **** (*p* < 0.0001).

Eryptosis is characterized by PS externalization which is detected via annexin-V-FITC staining. Figure 2b,c show that treatment with GAL at 300, 400, and 500 μM significantly increased the percentage of eryptotic cells (*p* < 0.0001). Next, we assessed hemolysis by measuring extracellular hemoglobin which was significantly increased (*p* < 0.0001) starting at 100 μM (Figure 2d). AST (Figure 2e), LDH (Figure 2f), and CK (Figure 2g) enzyme activities were also significantly elevated along with extracellular K^+^ levels (Figure 2h) confirming membrane damage. The two forms of cell death, as instigated by GAL, were, however, not correlated as shown via regression analysis (Figure 2i). Further examination of hemolysis under hypotonic conditions revealed instead that 10 μM of GAL protected RBCs against hypotonic lysis at 0.3% (*p* < 0.05) and 0.5% (*p* < 0.0001) NaCl (Figure 2j). AChE is an important marker of erythrocyte health as it preserves the cell membrane structure and morphology. It was revealed (Figure 2k) that GAL significantly inhibited AChE activity at 300 μM (*p* = 0.001), 400 μM, and 500 μM (*p* = 0.0001). While the role of vitamin B_12_ in RBC maturation is well-established, its function in mature erythrocytes remains ambiguous although it has been shown to serve as an antioxidant and a cofactor for a number of enzymes. Our results (Figure 2l) show that GAL significantly increased intracellular concentrations of vitamin B_12_ at 400 μM (*p* = 0.0137) and 500 μM (*p* = 0.0001) probably reflecting restricted utilization. Another hallmark of eryptosis is increased corpuscular aggregability which can be assessed by measuring the ESR (Figure 2m) which was indeed significantly increased upon exposure to 500 μM of GAL (*p* = 0.0161).

**Figure 3:**
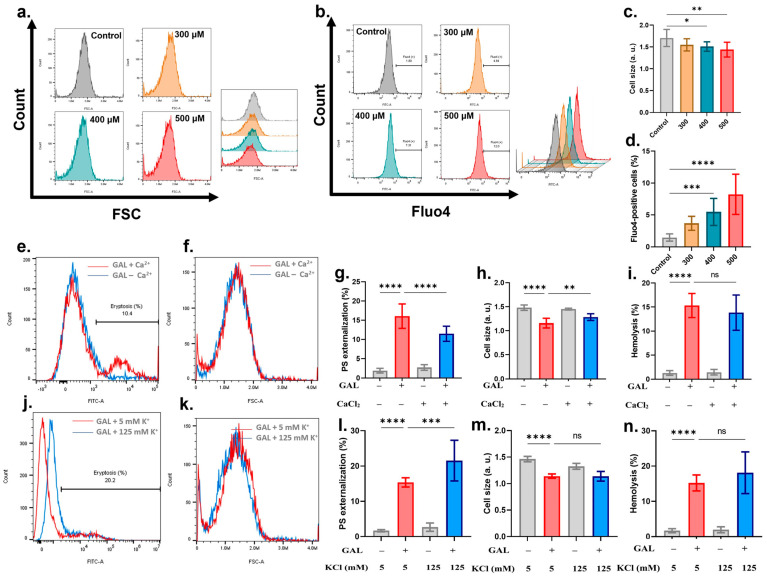
GAL causes loss of cellular volume and Ca^2+^ nucleation. (**a**) Original histograms of FSC signals. (**b**) Original histograms of Fluo4 fluorescence. (**c**) Percentage of cell shrinkage. (**d**) Percentage with increased Ca^2+^. (**e**) Original histograms of annexin-V-FITC with and without Ca^2+^. (**f**) Original histograms of FSC with and without Ca^2+^. (**g**) Percentage of eryptotic cells. (**h**) Percentage of cell shrinkage. (**i**) Percentage of hemolyzed cells. (**j**) Original histograms of annexin-V-FITC in 5 and 125 mM KCl. (**k**) Original histograms of FSC in 5 and 125 mM KCl. (**l**) Percentage of eryptotic cells. (**m**) Percentage of cell shrinkage. (**n**) Percentage of hemolyzed cells. Graphs show means ± SD. No significance is indicated by ns whereas * (*p* < 0.05), ** (*p* < 0.01), *** (*p* < 0.001), and **** (*p* < 0.0001).

**Figure 4:**
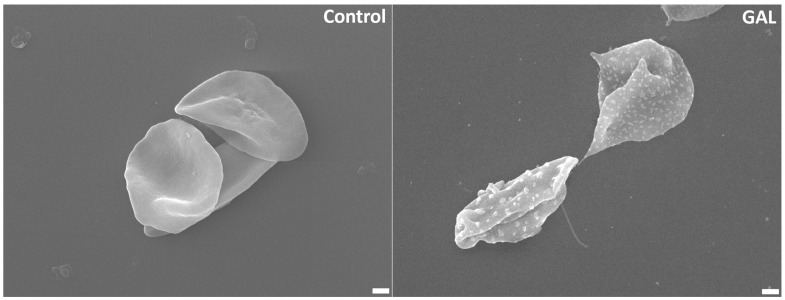
Ultrastructural morphology of RBCs. GAL induces the formation of granulated echinocytes. Magnification: ×5000. Scale bar: 1 μm.

Cell shrinkage follows PS externalization to expedite phagocytic removal of eryptotic cells. We assessed cell size via FSC and found that GAL significantly diminished cell size (Figure 3a,c) at 400 μM (*p* = 0.05) and 500 μM (*p* = 0.01). In an attempt to identify the mechanism behind the loss of cellular volume, cytosolic Ca^2+^, which is a major regulator of cellular volume, was measured. Figure 3b,d demonstrates significantly increased proportions of Fluo4-positive cells at 400 μM (*p* = 0.001) and 500 μM (*p* = 0.0001). After we established the role of Ca^2+^ in driving GAL-induced cell death, it was of interest to assess whether Ca^2+^ availability was required for GAL cytotoxicity. To this end, cells were incubated with and without 500 μM of GAL in standard and Ca^2+^-free Ringer buffers and eryptosis and hemolysis were assessed. It was revealed that both PS externalization (16.04 ± 3.17% to 11.46 ± 1.96%, *p* = 0.0001, Figure 3e,f) and cell shrinkage (1.15 ± 0.1 a.u. to 1.28 ± 0.07 a.u., *p* = 0.01, Figure 3g), but not hemolysis (Figure 3h), were significantly reduced when Ca^2+^ was not present. Then, we sought to determine the importance of KCl exit from cells in mediating GAL toxicity. Thus, cells were treated with 500 μM of GAL in standard (5 mM KCl) and K^+^-rich (125 mM KCl) Ringer buffers. Interestingly, Figure 3i,j shows that the eryptotic rate was significantly exacerbated by blocking KCl efflux (15.38 ± 1.30% to 21.56 ± 5.76%, *p* = 0.001) whereas FSC (Figure 3k) and hemolysis (Figure 3l) were unchanged. The observed cell shrinkage prompted us to further examine the ultrastructural morphology using SEM. As depicted in Figure 4, exposure to 500 μM of GAL resulted in echinocyte formation with prominent granulation.

**Figure 5:**
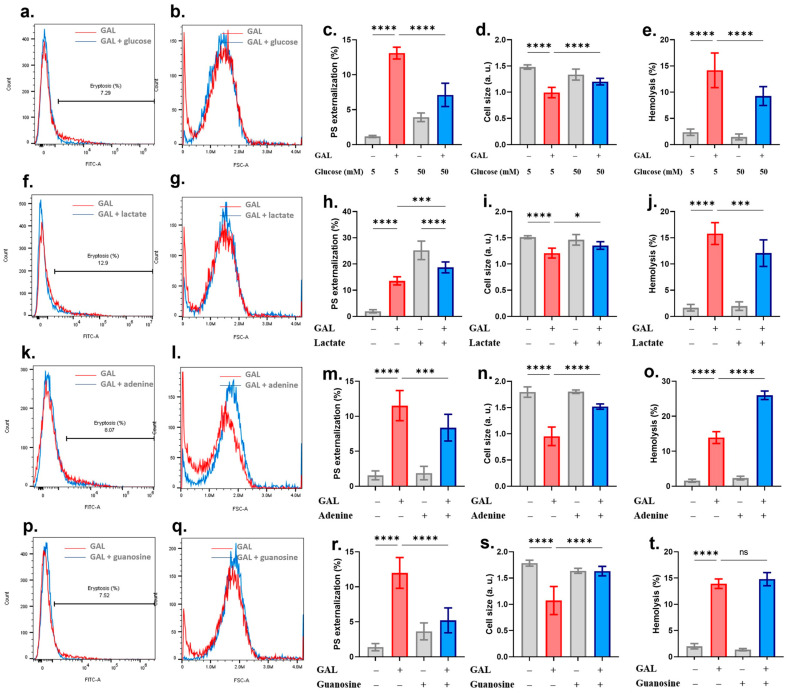
Energy replenishment reverses GAL-induced cytotoxicity. (**a**) Original annexin-V-FITC histograms in 5 and 50 mM glucose. (**b**) Original FSC histograms in 5 and 50 mM glucose. (**c**) Percentage of eryptotic cells. (**d**) Percentage of cell shrinkage. (**e**) Percentage of hemolyzed cells. (**f**) Original annexin-V-FITC histograms with and without lactate. (**g**) Original FSC histograms with and without lactate. (**h**) Percentage of eryptotic cells. (**i**) Percentage of cell shrinkage. (**j**) Percentage of hemolyzed cells. (**k**) Original annexin-V-FITC histograms with and without adenine. (**l**) Original FSC histograms with and without adenine. (**m**) Percentage of eryptotic cells. (**n**) Percentage of cell shrinkage. (**o**) Percentage of hemolyzed cells. (**p**) Original annexin-V-FITC histograms with and without guanosine. (**q**) Original FSC histograms with and without guanosine. (**r**) Percentage of eryptotic cells. (**s**) Percentage of cell shrinkage. (**t**) Percentage of hemolyzed cells. Graphs show means ± SD. No significance is indicated by ns whereas * (*p* < 0.05), *** (*p* < 0.001) and **** (*p* < 0.0001).

Next, we evaluated the effect of glucose, lactate, adenine, and guanosine on GAL toxicity since energy exhaustion invariably promotes cell death. In this set of experiments, cells were co-incubated with 500 μM of GAL and 50 mM of glucose. We found that glucose loading significantly (*p* < 0.0001) inhibited PS exposure (Figure 5a,c), shrinkage (Figure 5b,d), and hemolysis (Figure 5e). Interestingly, 28 mM lactate, on the other hand, stimulated eryptosis on its own (Figure 5f,h) which was significantly ameliorated in the presence of GAL (*p* < 0.0001). However, similar to glucose, lactate prevented both GAL-induced cell shrinkage (Figure 5g,i, *p* < 0.05) and hemolysis (Figure 5j, *p* < 0.0001). Furthermore, cells were exposed to GAL in the presence of 2 mM of adenine or guanosine. While adenine significantly diminished GAL-induced PS exposure (Figure 5k,m, *p* < 0.001) and cell shrinkage (Figure 5l,n, *p* < 0.0001), it exacerbated hemolysis (Figure 5o, *p* < 0.0001). On the contrary, guanosine significantly alleviated PS exposure (Figure 5p,r, *p* < 0.0001) and cell shrinkage (Figure 5q,s, *p* < 0.0001) without affecting hemolysis (Figure 5t).

**Figure 6:**
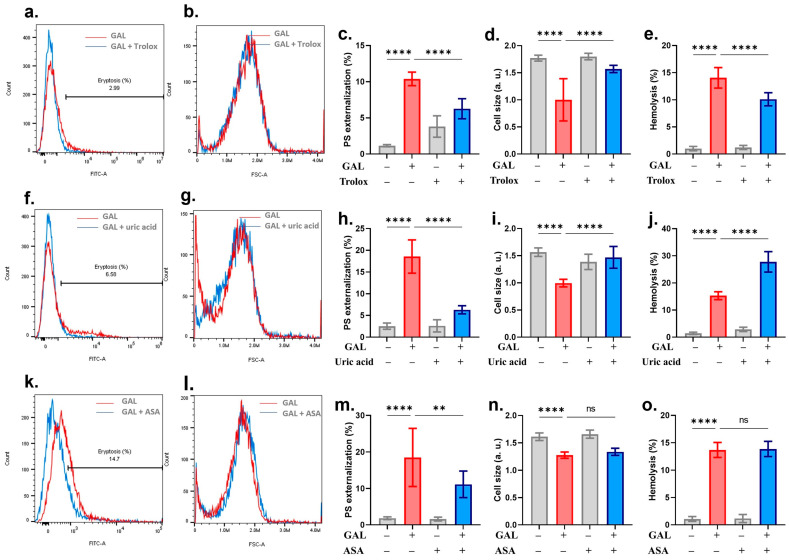
Antieryptotic and antihemolytic effects of Trolox, uric acid, and ASA. (**a**) Original annexin-V-FITC histograms with and without Trolox. (**b**) Original FSC histograms with and without Trolox. (**c**) Percentage of eryptotic cells. (**d**) Percentage of cell shrinkage. (**e**) Percentage of hemolyzed cells. (**f**) Original annexin-V-FITC histograms with and without uric acid. (**g**) Original FSC histograms with and without uric acid. (**h**) Percentage of eryptotic cells. (**i**) Percentage of cell shrinkage. (**j**) Percentage of hemolyzed cells. (**k**) Original annexin-V-FITC histograms with and without ASA. (**l**) Original FSC histograms with and without ASA. (**m**) Percentage of eryptotic cells. (**n**) Percentage of cell shrinkage. (**o**) Percentage of hemolyzed cells. Graphs show means ± SD. No significance is indicated by ns whereas ** (*p* < 0.01) and **** (*p* < 0.0001).

**Figure 7:**
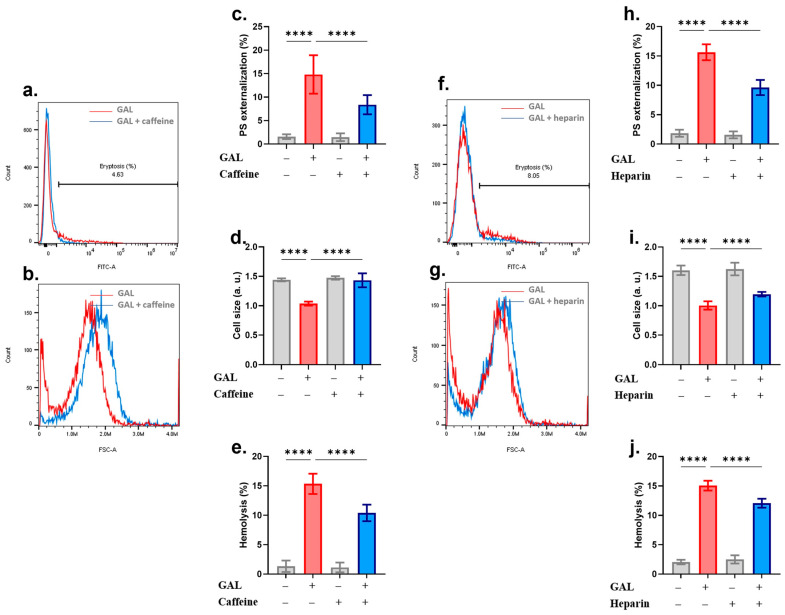
Ameliorative effects of caffeine and heparin. (**a**) Original annexin-V-FITC histograms with and without caffeine. (**b**) Original FSC histograms with and without caffeine. (**c**) Percentage of eryptotic cells. (**d**) Percentage of cell shrinkage. (**e**) Percentage of hemolyzed cells. (**f**) Original annexin-V-FITC histograms with and without heparin. (**g**) Original FSC histograms with and without heparin. (**h**) Percentage of eryptotic cells. (**i**) Percentage of cell shrinkage. (**j**) Percentage of hemolyzed cells. Graphs show means ± SD **** (*p* < 0.0001).

The roles of Trolox, uric acid, and ASA in mitigating GAL toxicity were also assessed. Figure 6a–e shows that while Trolox rescued the cells from both GAL-induced eryptosis and hemolysis, uric acid, although inhibiting eryptosis, aggravated hemolysis (Figure 6f–j). The only endpoint significantly ameliorated by ASA was PS exposure (Figure 6k,m). Furthermore, caffeine [17] and heparin [18] have been identified as inhibitors of eryptosis. Our findings in Figure 7 corroborate these reports as both compounds significantly ameliorated the hallmarks of eryptosis: PS exposure and cell shrinkage, as well as hemolysis. 

**Figure 8:**
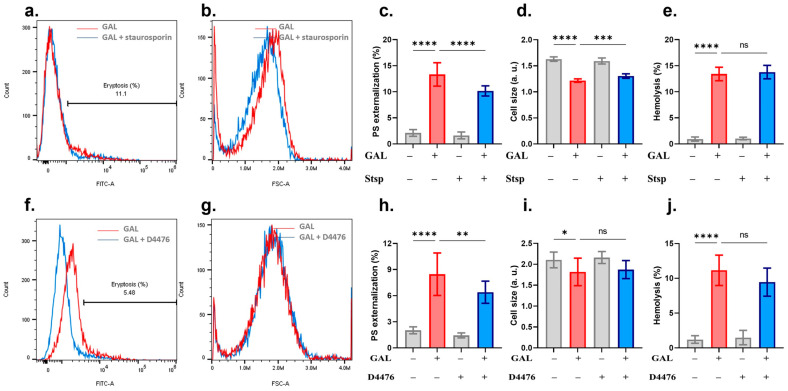
Inhibitors of GAL-induced eryptosis. (**a**) Original annexin-V-FITC histograms with and without staurosporin. (**b**) Original FSC histograms with and without staurosporin. (**c**) Percentage of eryptotic cells. (**d**) Percentage of cell shrinkage. (**e**) Percentage of hemolyzed cells. (**f**) Original annexin-V-FITC histograms with and without D4476. (**g**) Original FSC histograms with and without D4476. (**h**) Percentage of eryptotic cells. (**i**) Percentage of cell shrinkage. (**j**) Percentage of hemolyzed cells. Graphs show means ± SD. No significance is indicated by ns whereas * (*p* < 0.05), ** (*p* < 0.01), *** (*p* < 0.001), and **** (*p* < 0.0001).

**Figure 9:**
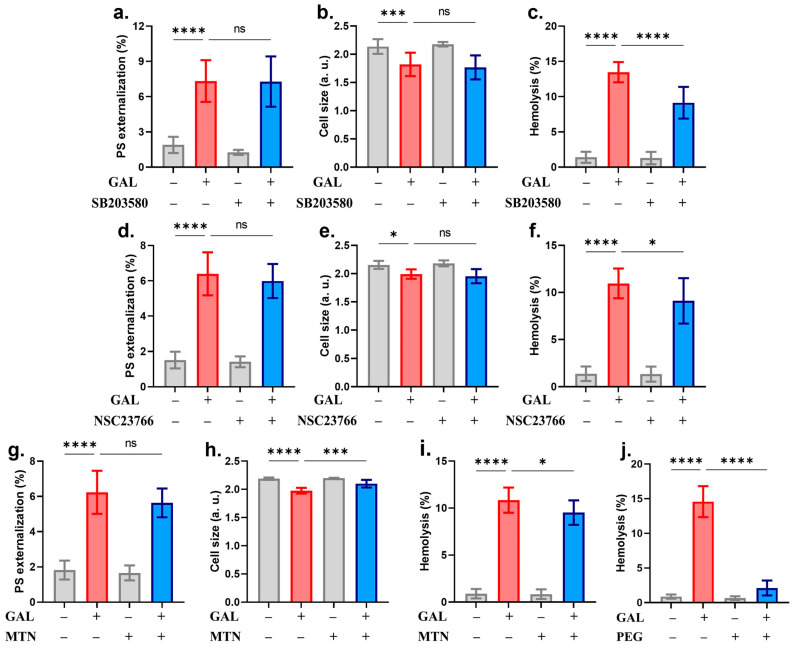
Inhibitors of GAL-induced hemolysis. (**a**) Percentage of eryptotic cells, (**b**) shrinkage, and (**c**) hemolysis with and without SB203580. (**d**) Percentage of eryptotic cells, (**e**) shrinkage, and (**f**) hemolysis with and without NSC23766. (**g**) Percentage of eryptotic cells, (**h**) shrinkage, and (**i**) hemolysis with and without MTN. (**j**) Effect of GAL on hemolysis with and without PEG. Graphs show means ± SD. No significance is indicated by ns whereas * (*p* < 0.05), *** (*p* < 0.001), and **** (*p* < 0.0001).

**Figure 10:**
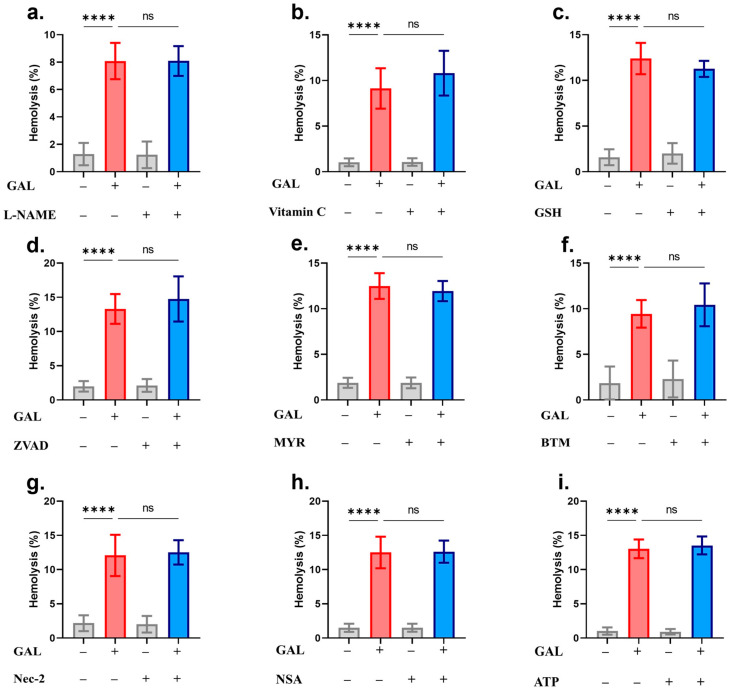
Analysis of antioxidants and signaling pathways. Hemolytic rates in the presence and absence of (**a**) L-NAME, (**b**) vitamin C, (**c**) GSH, (**d**) Z-VAD-FMK, (**e**) myriocin, (**f**) BAPTA-AM, (**g**) necrostatin-2, (**h**) NSA, and (**i**) ATP. Graphs show means ± SD. No significance is indicated by ns whereas **** (*p* < 0.0001).

Analysis of signaling pathways using small-molecule inhibitors identified both PKC and CK1α as essential modulators of GAL-induced eryptosis. Figure 8a–e shows that PS exposure and cell shrinkage but not hemolysis were significantly decreased in the presence of staurosporin. However, blocking CK1α activity with D4476 could only alleviate PS exposure with no appreciable effect on either cell shrinkage or hemolysis (Figure 8f–j). Instead, the hemolytic potential of GAL was significantly diminished in the presence of SB203580 (Figure 9c), NSC23766 (Figure 9f), MTN (Figure 9i), and completely abrogated by PEG (14.58 ± 2.23% to 2.11 ± 1.07%, *p* = 0.0001, Figure 9j). All other antioxidants and inhibitors used were unable to provide significant effects (Figure 10).

**Figure 11:**
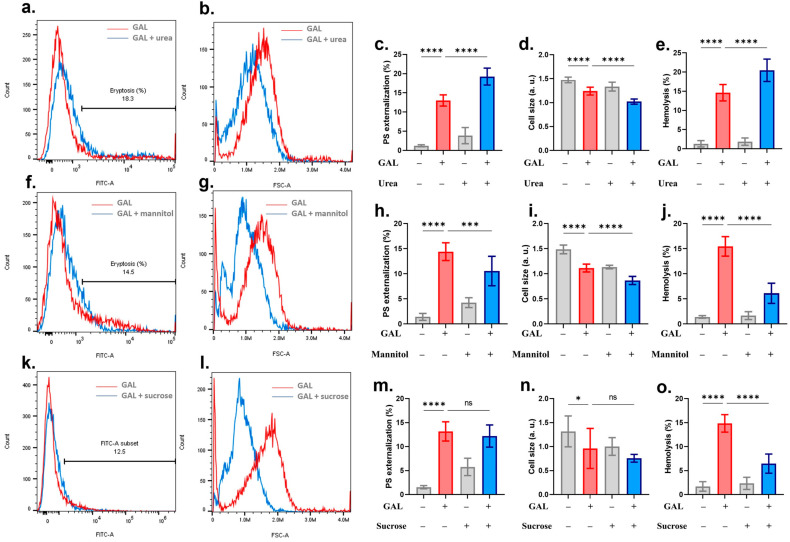
GAL toxicity under hyperosmotic stress. (**a**) Original annexin-V-FITC histograms with and without urea. (**b**) Original FSC histograms with and without urea. (**c**) Percentage of eryptotic cells. (**d**) Percentage of cell shrinkage. (**e**) Percentage of hemolyzed cells. (**f**) Original annexin-V-FITC histograms with and without mannitol. (**g**) Original FSC histograms with and without mannitol. (**h**) Percentage of eryptotic cells. (**i**) Percentage of cell shrinkage. (**j**) Percentage of hemolyzed cells. (**k**) Original annexin-V-FITC histograms with and without sucrose. (**l**) Original FSC histograms with and without sucrose. (**m**) Percentage of eryptotic cells. (**n**) Percentage of cell shrinkage. (**o**) Percentage of hemolyzed cells. Graphs show means ± SD. No significance is indicated by ns whereas * (*p* < 0.05), *** (*p* < 0.001), and **** (*p* < 0.0001).

Hyperosmotic stress has been reported to trigger both eryptosis and hemolysis. The potential additive effect of GAL on osmotic challenge induced by urea, sucrose, and mannitol was thus investigated. GAL significantly (*p* = 0.0001) aggravated urea-induced PS exposure, cell shrinkage, and hemolysis (Figure 11a–e) while only mannitol-induced cell shrinkage was augmented by GAL (Figure 11f–j). With regard to PS exposure and hemolysis, mannitol exhibited a rather protective role, an observation mirrored by sucrose only in the case of hemolysis (Figure 11k–o).

## 3. Discussion

In this work, we established a novel activity of GAL which is the stimulation of eryptosis and hemolysis in human erythrocytes. Since premature RBC death is implicated in chemotherapy-related anemia, the findings disclosed herein inform future research directions regarding the clinical prospects of GAL as a potential anticancer therapeutic.

Externalization of PS occurs due to the disrupted asymmetrical arrangement of the cell membrane phospholipids to aid in efferocytosis through PS receptors on phagocytes. When cells are prematurely eliminated from the circulation, the bone marrow becomes unable to sufficiently upregulate erythropoiesis, leading to anemia due to an appreciable decline in circulating RBCs. Eryptotic cells also tend to aggregate and promote thromboembolic events due to the high affinity of exposed PS molecules to the vascular endothelium, evidenced in this study by the elevated ESR (Figure 2). In addition to its recognized role in chemotherapy-related anemia, eryptosis similarly participates in a growing number of conditions such as diabetes mellitus, renal failure, sepsis, and cancer [19]. In particular, acute erythroid leukemia presents with severe hemolytic anemia characterized by low RBCs and hemoglobin, reticulocytosis, and elevated LDH and bilirubin [20]. Therefore, the clinical utility of GAL in this vulnerable patient group must be cautiously approached. Specifically, the identified inhibitors of GAL toxicity may be invaluable to enhance its specificity to target cancer cells.

The execution of eryptosis involves numerous underlying mechanisms among which Ca^2+^ signaling seems to be the most important. Our results indicate that GAL requires Ca^2+^ entry for its full eryptotic activity (Figure 3) as the absence of extracellular Ca^2+^ significantly blunted both PS externalization and cell shrinkage. GAL, on the other hand, does not seem to rely on the activity of Ca^2+^ channels to execute hemolysis. This is comprehensible considering that the movement of phospholipids within the membrane bilayer is contingent upon the activity of Ca^2+^-dependent enzymes [21]. As such, the deranged regulation of Ca^2+^ invariably modulates the activity of these enzymes culminating in loss of membrane asymmetry. Notably, Ca^2+^ channels may also be activated by cyclooxygenases (COX) [22], and the reversal of PS exposure we observed in the presence of ASA (Figure 6) further implicates these enzymes in driving GAL-induced eryptosis. Interestingly, it has been demonstrated that stressed erythrocytes release microvesicles upon Ca^2+^ accumulation that are rich in AChE [23]. Thus, the reduced AChE activity we detected upon GAL treatment (Figure 2) may be related to the increased Ca^2+^ levels and the subsequent loss of AChE in the membrane.

Another consequence of Ca^2+^ nucleation within the cytoplasm is its ability to activate Ca^2+^-responsive K^+^ channels. When these channels are inordinately activated, exaggerated release of KCl and water follows. It is this cellular dehydration that is responsible for the observed shrinkage typical of eryptosis (Figure 3). Upon further inspection of the cellular morphology using SEM, we found that GAL induced the formation of granulated echinocytes (Figure 4) indicating altered cytoskeleton proteins. Indeed, chemical stress has been shown to induce a variety of misshapen cells, and in this particular case, the appearance of granules may reflect severe membrane damage and the presence of denatured proteins. It remains to be seen, however, what membrane proteins are particularly affected by GAL.

In addition to eryptosis, this report identified GAL as a hemolytic agent (Figure 2). This is relevant as prompt recognition and correction of anemia in cancer patients has been shown to prolong survival. Chemotherapy-induced anemia can be caused either by suppression of erythropoiesis or by hemolysis of circulating cells. Bone marrow suppression and diminished erythropoietin production due to nephrotoxicity have long been recognized as important mechanisms underlying the decreased RBC production seen in patients [24]. As for hemolysis, three forms have been characterized; namely microangiopathic hemolytic anemia, immune hemolytic anemia, and oxidative hemolysis. Microangiopathic hemolysis occurs due to the physical compression of erythrocytes within the microvasculature due to the presence of microthrombi and endothelial injury. This type has a mortality rate of 50% and is associated with thrombus formation whose risk increases with eryptosis [25]. Therefore, being a pro-eryptotic agent, it seems likely that GAL may cause hemolytic anemia in patients through this mechanism. This is comprehensible considering that evidence for the formation of GAL-related antibodies that mimic autoimmune hemolysis has yet to be demonstrated [26], whereas oxidative hemolysis does not appear to be relevant as GAL cytotoxicity was independent of ROS formation (Figure S1) which is in agreement with its established antioxidant activity [27]. Notably, the lack of correlation between eryptosis and hemolysis (Figure 2) along with the varying effects of different modulators on both types leaves virtually no doubt that they are two biochemically distinct cell death modalities.

The strength of this study lies in the wide assortment of compounds and small molecule modulators employed to dissect the biochemical mechanisms that mediate GAL action in RBCs (Table 1).

First, we demonstrated that increasing glucose concentration from 5 mM to 50 mM served to protect the cells against eryptosis and hemolysis (Figure 5). Viskupicova et al. [28] reported that, compared to cells suspended in 5 mM of glucose, those cultured in 45 mM of glucose showed significantly increased hemoglobin glycation, catalase activity, and GSH and GSSG levels as well as diminished eryptosis and glutathione-*S*-transferase and glutathione reductase activities. Metabolic replenishment through glucose, adenine, and guanosine invariably prevents ATP depletion and bioenergetic catastrophe which is characteristic of eryptosis [29]. This is because a constant supply of energy is pivotal to cell survival as it is required for the maintenance of membrane asymmetry, ion trafficking, and metabolic processes. In fact, PS externalization has been shown to be triggered by Janus-activated kinase 3 (JAK3) which is activated during metabolic exhaustion [30]. Therefore, JAK3 may mediate GAL-induced eryptosis through ATP depletion.

In fact, eryptosis has been shown to be significantly increased in diabetics compared to healthy subjects [31]. Therefore, it is expected that GAL toxicity is augmented in these vulnerable patients who already have higher baseline eryptotic rates. This might be related to the low-grade chronic inflammation characteristic of diabetes especially considering that the duration of diabetes increases eryptosis. It is possible that long-term exposure to sustained hyperglycemia might be harmful while short-term treatment, as in our study, is rather beneficial. Future studies comparing eryptosis rates in healthy subjects and patients with controlled and uncontrolled diabetes are likely to reveal whether hyperglycemia itself or the associated metabolic alterations account for the higher eryptotic rate.

Adenine and guanosine are other important energy substrates that, similar to glucose, rescued the cells from eryptosis but had no inhibitory effect on hemolysis (Figure 5). Adenosine has previously been shown to inhibit eryptosis in part by blocking Ca^2+^ influx [32]. Since Ca^2+^ is essential for the full eryptotic but not hemolytic activity of GAL (Figure 3), it is highly likely that adenine and guanosine also target Ca^2+^ signaling to exert their protective effects. The current findings suggest that either utilization of energy precursors is disrupted by GAL or that activation of glycolysis is required to counteract the hemolytic activity of GAL. This latter hypothesis is corroborated by the fact that lactate, a product of glycolysis under anaerobic conditions, ameliorates the effect of GAL on cell shrinkage and hemolysis (Figure 5). Lactate at this concentration (28 mM) is added to lactated Ringer’s injection used for fluid replacement to counteract changes in pH and to preserve electrolyte homeostasis. Thus, the mitigating effects of lactate on GAL toxicity could also be related to these mechanisms. The fact that GAL alleviated lactate-induced eryptosis argues for the antioxidant role previously shown for GAL [27].

Caffeine has been demonstrated to possess antieryptotic properties by counteracting Ca^2+^ elevations induced by metabolic exhaustion [17]. Heparin has similarly been used to inhibit the eryptotic activity of extracellular histones [18,33] although the underlying mechanisms have largely been overlooked. In the case of GAL, both caffeine and heparin partially reversed PS exposure, cell shrinkage, and hemolysis (Figure 6) possibly implicating Ca^2+^ as a mediator in these processes. Nonetheless, further investigation into the mechanisms through which caffeine and heparin prolong RBC survival is recommended.

Trolox is a vitamin E analog employed as a ROS scavenger to study the involvement of oxidative damage to toxic endpoints. It is rather surprising that Trolox was effective in blocking GAL toxicity (Figure 7) although no oxidative damage was observed (Figure S1). This potentially points to a functional role of Trolox unrelated to ROS neutralization, such as the modulation of Ca^2+^ channel activity [34], which has also been shown for vitamin C [35]. A similar antihemolytic effect was observed for MTN (Figure 9) which, based on current evidence, is unlikely to be ascribed to its antioxidant activity. Instead, MTN acts as an anti-inflammatory agent [36] and its effect may therefore mimic that of ASA (Figure 7).

Although the apoptotic effects of uric acid have been reported at 0.1 to 0.7 mM [37,38], our results show that 1 mM of uric acid significantly blunted PS externalization and cell shrinkage but augmented the hemolytic activity of GAL (Figure 7). To the best of our knowledge, this is the first demonstration of the antieryptotic role of uric acid in the literature, and it could very well be related to its antioxidant activity as seen in previous studies [39,40]. Uric acid can also act as a pro-oxidant and promote apoptosis [40,41] which explains its enhancing effect on hemolysis. This dichotomy emphasizes the cell- and pathway-specific contexts in which compounds function. Notably, reversine and volasertib, among others, have both been reported to prevent eryptosis despite stimulating apoptosis in nucleated cells [42,43].

We analyzed the involvement of a host of signal transduction enzymes and found that PKC and CK1α mediate the eryptotic activity of GAL (Figure 8) whereas p38 MAPK and Rac1 mediate its hemolytic activity (Figure 9). During eryptosis, PKC and CK1α are activated upon metabolic exhaustion [44,45] which we have identified as an essential mechanism targeted by GAL to bring about both forms of erythrocyte death (Figure 5). Thus, PKC and CK1α seem to act upstream of ATP depletion. On the other hand, p38 MAPK has been shown to be phosphorylated to mediate erythrocyte death in osmotically stressed cells [46,47] and in response to toxic compounds [48,49,50]. Unlike the fairly established role of the previous enzymes in RBC death, the participation of Rac1 GTPase has only recently emerged. Attanzio et al. [51] reported that oxysterols require Rac1 GTPase activation to cause ROS-induced eryptosis. However, the exact mechanisms through which Rac1 mediate eryptosis remain unexplored. In the apoptosis of nucleated cells, Rac1 is known to be concomitantly activated with ceramide and Fas, both of which signal for erythrocyte death [49,52]. It follows then that their involvement in the signaling pathway of Rac1 that mediates GAL-induced hemolysis is highly likely.

We subjected GAL-treated erythrocytes to hyperosmotic conditions using urea, mannitol, and sucrose (Figure 11) and a few unique observations merit comment. In the case of urea, a synergistic effect with GAL was noted for all toxic endpoints including PS externalization, cell shrinkage, and hemolysis, suggesting that urea may exacerbate the toxic effects of GAL particularly in vulnerable cohorts such as those with kidney disease [53]. Importantly, urea has been shown to stimulate cation channel activity [54] and to trigger eryptosis [55] which could be responsible for its synergistic effect with GAL. In contrast, mannitol blunted membrane scrambling as well as hemolysis but enhanced volume loss. RBC storage solutions are supplemented with mannitol to minimize hemolysis [56]. Also, being a non-penetrating compound, mannitol generates an osmotic gradient that forces water out of the cells leading to crenation which is aggravated by GAL. Although conclusions regarding its protective effects cannot be safely drawn based on available evidence, it is speculated that mannitol interferes with the cellular machinery targeted by GAL, most notably Ca^2+^ signaling (Figure 3) and ATP depletion (Figure 5). Lastly, sucrose induces PS externalization through ceramide and Ca^2+^ signaling [54] but its effect on eryptosis in the presence of GAL was unremarkable. Instead, sucrose was effective in abrogating GAL-induced hemolysis which strongly points to the inhibition of water influx as sucrose increases the osmotic pressure of the medium.

## 4. Materials and Methods

### 4.1. Blood Collection

This study was approved by the Ethics Committee of King Saud University Medical City (E-23-7764). All donors signed an informed consent form in line with the Declaration of Helsinki. A total of 21 donors, 13 males and 8 females ranging from 23 to 36 years of age with normal complete blood count (CBC) and body mass index (BMI) results, took part in the study. Blood samples were acquired via venipuncture in lithium heparin and processed within 1 h of collection. Aliquots were washed three times in PBS and 30% packed cell suspensions were finally prepared in Ca^2+^-free Ringer buffer [57].

### 4.2. Chemicals, Reagents, and Experimental Design

Chemicals and reagents were procured from Solarbio Life Science (Beijing, China). GAL was prepared in DMSO as a 50 mM stock solution (13.5 mg/mL) and stored at −80 °C. To individual Ringer solutions, urea (150 mM), sucrose (280 mM), glucose (50 mM), mannitol (284 mM), caffeine (0.5 mM), adenine (2 mM), guanosine (2 mM), uric acid (1 mM), Trolox (50 μM), or L-lactate (28 mM) was added. In separate sets of experiments, RBCs were co-treated with 500 μM of GAL with or without 10 μM of BAPTA-AM (cell-permeable Ca^2+^ chelator), 100 μM of Z-VAD-FMK (pan-caspase inhibitor), 100 μM of SB203580 (p38 inhibitor), 20 μM of D4476 (CK1α inhibitor), 1 μM of staurosporin (PKC inhibitor), 100 μM of NSC23766 (Rac1 GTPase inhibitor), 100 μM of necrostatin-2 (receptor-interacting protein 1 inhibitor), 0.5 μM of necrosulfonamide (NSA; mixed lineage kinase domain like pseudokinase inhibitor), 20 μM of L-NAME (nitric oxide synthase inhibitor), 1 mM of vitamin C, 50 μM of acetylsalicylic acid (ASA), 20 μM of reduced glutathione (GSH), 50 μM of melatonin (MTN), or 10 μM of myriocin (serine palmitoyltransferase inhibitor). The study design is shown in Figure 1.

### 4.3. Eryptotic Markers

Cells were stained with annexin-V-FITC, Fluo4/AM, and 2′,7′-dichlorodihydrofluorescein diacetate (H_2_DCFDA) to analyze cell membrane scrambling, Ca^2+^, and ROS, respectively, using a Northern Lights flow cytometer (Cytek Biosciences, Fremont, CA, USA). In brief, 50 μL of RBCs were added to 150 μL of Ringer solution containing 5 mM CaCl_2_ and 1% annexin-V-FITC, 2 μM Fluo4/AM, or 5 μM H_2_DCFDA. Cells were examined for annexin-V binding following incubation for 10 min at room temperature, and for Fluo4 and DCF after incubation for 30 min at 37 °C. Cell size was inferred from the forward scatter channel (FSC) signal expressed in arbitrary units (a. u.) [58]. The erythrocyte sedimentation rate (ESR) was determined in Westergren tubes as a function of cellular aggregation [59].

### 4.4. Hemolytic Markers

Supernatants were harvested via centrifugation (13,000× *g*, 1 min) to detect extracellular hemoglobin at 405 nm (LMPR-A14 microplate reader, Labtron Equipment Ltd., Surrey, UK) and hemolysis was expressed relative to distilled water suspensions [60]. CK, AST, LDH, Mg^2+^, and K^+^ were assayed in the supernatants via a DxC 700 AU chemistry analyzer (Beckman Coulter, Pasadena, CA, USA) using colorimetric and ion-selective electrode methods.

### 4.5. AChE Activity

AChE activity was measured via Ellman’s assay using an AChE Activity Assay Kit (Solarbio) [60]. This two-step assay is based on the hydrolytic cleavage of acetylthiocholine into acetate and thiocholine by AChE and the subsequent reduction of 5,5′-dithiobis(2-nitrobenzoic acid) (DTNB) by thiocholine into 5-thio-2-nitrobenzoic acid (TNB). The absorbance of TNB at 412 nm is proportional to AChE activity.

### 4.6. RBC Aggregation

The ESR was determined in millimeters over one hour in Westergren tubes as a function of cellular aggregability [59].

### 4.7. Scanning Electron Microscopy (SEM)

Cell imaging was carried out with a JSM-7610F ultra-high-resolution Schottky field emission scanning electron microscope at 15.0 kV (JEOL Co., Ltd., Akishima, Tokyo, Japan) as detailed elsewhere [21]. Cell fixation was achieved with 2.5% glutaraldehyde, staining with 1% osmium tetroxide, and dehydration with 50–100% ethanol solutions.

### 4.8. B_12_

Intracellular B_12_ concentration was measured via a Beckman’s UniCel DxI 800 Access Immunoassay System analyzer.

### 4.9. Statistical Analysis

All parameters were measured in triplicate samples taken from three experiments (*n* = 9). Student’s *t*-test and one-way ANOVA were applied for the comparison of means as appropriate. The null hypothesis was rejected when the *p* value was less than 0.05 as calculated by Prism 9.0 (GraphPad Software, Inc., San Diego, CA, USA).

## 5. Conclusions

**Figure 12:**
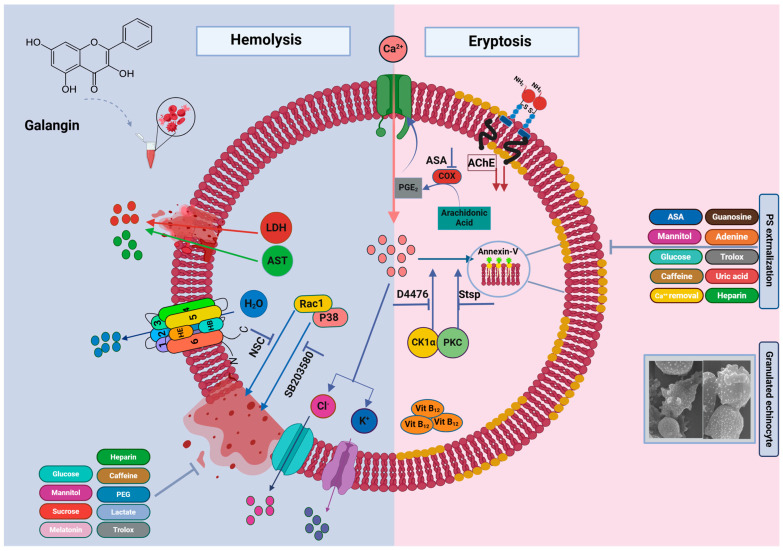
A working model of GAL-induced RBC death. Prepared with BioRender.

In conclusion, this work shows that GAL triggers concurrent eryptosis and hemolysis through Ca^2+^ nucleation, metabolic collapse, and anticholinesterase activity. Eryptosis was mediated through COX, PKC, and CK1α whereas hemolysis required the activity of p38 MAPK and Rac1 GTPase; both modalities were inhibited by glucose, heparin, caffeine, Trolox, and mannitol. While Ca^2+^ elimination, adenine, guanosine, ASA, and uric acid were only effective in reversing PS externalization, lactate, MTN, PEG, and sucrose solely prevented hemolysis (Figure 12).

## Figures and Tables

**Table 1:** Summary of GAL-induced RBC death.

Inhibitor	PS Externalization	Cell Shrinkage	Hemolysis
Glucose	+	+	+
Trolox	+	+	+
Caffeine	+	+	+
Heparin	+	+	+
Ca^2+^ removal	+	+	−
Adenine	+	+	−
Guanosine	+	+	−
Uric acid	+	+	−
Staurosporin	+	+	−
Mannitol	+	−	+
Lactate	−	+	+
Melatonin	−	+	+
Acetylsalicylic acid	+	−	−
D4476	+	−	−
Sucrose	−	−	+
Polyethylene glycol 8000	−	−	+
SB203580	−	−	+
NSC23766	−	−	+

“+” indicates prevention of toxic endpoint. “−” indicates lack of inhibition.

## Data Availability

The data that support the findings of this study are available on request from the corresponding author.

## Supplementary Materials

The following supporting information can be downloaded at: https://www.mdpi.com/article/10.3390/ijms252212267/s1.

## References

[1] Suvarna V., Gujar P., Murahari M. (2017). Complexation of phytochemicals with cyclodextrin derivatives—An insight. Biomed. Pharmacother..

[2] Zhang H.T., Wu J., Wen M., Su L.J., Luo H. (2012). Galangin induces apoptosis in hepatocellular carcinoma cells through the caspase 8/t-Bid mitochondrial pathway. J. Asian Nat. Prod. Res..

[3] Zhang H.T., Luo H., Wu J., Lan L.B., Fan D.H., Zhu K.D., Chen X.Y., Wen M., Liu H.M. (2010). Galangin induces apoptosis of hepatocellular carcinoma cells via the mitochondrial pathway. World J. Gastroenterol..

[4] Zhong X., Huang S., Liu D., Jiang Z., Jin Q., Li C., Da L., Yao Q., Wang D. (2020). Galangin promotes cell apoptosis through suppression of H19 expression in hepatocellular carcinoma cells. Cancer Med..

[5] Kim D.A., Jeon Y.K., Nam M.J. (2012). Galangin induces apoptosis in gastric cancer cells via regulation of ubiquitin carboxy-terminal hydrolase isozyme L1 and glutathione S-transferase P. Food Chem. Toxicol..

[6] Cao J., Wang H., Chen F., Fang J., Xu A., Xi W., Zhang S., Wu G., Wang Z. (2016). Galangin inhibits cell invasion by suppressing the epithelial-mesenchymal transition and inducing apoptosis in renal cell carcinoma. Mol. Med. Rep..

[7] Han M.A., Lee D.H., Woo S.M., Seo B.R., Min K.J., Kim S., Park J.-W., Kim S.H., Choi Y.H., Kwon T.K. (2016). Galangin sensitizes TRAIL-induced apoptosis through down-regulation of anti-apoptotic proteins in renal carcinoma Caki cells. Sci. Rep..

[8] Liu D., You P., Luo Y., Yang M., Liu Y. (2018). Galangin Induces Apoptosis in MCF-7 Human Breast Cancer Cells Through Mitochondrial Pathway and Phosphatidylinositol 3-Kinase/Akt Inhibition. Pharmacology.

[9] Lee C.C., Lin M.L., Meng M., Chen S.S. (2018). Galangin Induces p53-independent S-phase Arrest and Apoptosis in Human Nasopharyngeal Carcinoma Cells Through Inhibiting PI3K-AKT Signaling Pathway. Anticancer Res..

[10] Huang H., Chen A.Y., Ye X., Guan R., Rankin G.O., Chen Y.C. (2020). Galangin, a Flavonoid from Lesser Galangal, Induced Apoptosis via p53-Dependent Pathway in Ovarian Cancer Cells. Molecules.

[11] Kong Y., Feng Z., Chen A., Qi Q., Han M., Wang S., Zhang Y., Zhang X., Yang N., Wang J. (2019). The Natural Flavonoid Galangin Elicits Apoptosis, Pyroptosis, and Autophagy in Glioblastoma. Front. Oncol..

[12] Long X., Chen L., Yang J., Dong T., Cheng Q., Wang W., Zou Y., Su Y., Dai W., Chen B. (2023). Network-based Pharmacology and In vitro Validation Reveal that Galangin Induces Apoptosis in Bladder Cancer Cells by Promoting the P53 Signaling Pathway. Anticancer Agents Med. Chem..

[13] Birgegård G., Henry D., Glaspy J., Chopra R., Thomsen L.L., Auerbach M. (2016). A Randomized Noninferiority Trial of Intravenous Iron Isomaltoside versus Oral Iron Sulfate in Patients with Nonmyeloid Malignancies and Anemia Receiving Chemotherapy: The PROFOUND Trial. Pharmacotherapy.

[14] Lang E., Bissinger R., Qadri S.M., Lang F. (2017). Suicidal death of erythrocytes in cancer and its chemotherapy: A potential target in the treatment of tumor-associated anemia. Int. J. Cancer.

[15] Bettiol A., Galora S., Argento F.R., Fini E., Emmi G., Mattioli I., Bagni G., Fiorillo C., Becatti M. (2022). Erythrocyte oxidative stress and thrombosis. Expert Rev. Mol. Med..

[16] Dreischer P., Duszenko M., Stein J., Wieder T. (2022). Eryptosis: Programmed Death of Nucleus-Free, Iron-Filled Blood Cells. Cells.

[17] Floride E., Föller M., Ritter M., Lang F. (2008). Caffeine inhibits suicidal erythrocyte death. Cell. Physiol. Biochem..

[18] Yeung K.W., Lau P.M., Tsang H.L., Ho H.P., Kwan Y.W., Kong S.K. (2019). Extracellular Histones Induced Eryptotic Death in Human Erythrocytes. Cell. Physiol. Biochem..

[19] Alghareeb S.A., Alfhili M.A., Fatima S. (2023). Molecular Mechanisms and Pathophysiological Significance of Eryptosis. Int. J. Mol. Sci..

[20] Ota S., Kasahara A., Mizuno S., Uchikoga O., Kuroda M., Miyoshi H., Shiomi K., Umena S., Noguchi T., Kishimoto N. (2013). Two cases of acute erythroid leukemia presenting with marked macrocytic anemia, reticulocytosis and hemolysis. Intern. Med..

[21] Pretorius E., Du Plooy J.N., Bester J. (2016). A Comprehensive Review on Eryptosis. Cell. Physiol. Biochem..

[22] Lang P.A., Kempe D.S., Myssina S., Tanneur V., Birka C., Laufer S., Lang F., Wieder T., Huber S.M. (2005). PGE_2_ in the regulation of programmed erythrocyte death. Cell Death Differ..

[23] Leal J.K.F., Adjobo-Hermans M.J.W., Brock R., Bosman G.J.C.G.M. (2017). Acetylcholinesterase provides new insights into red blood cell ageing in vivo and in vitro. Blood Transfus..

[24] Abdel-Razeq H., Hashem H. (2020). Recent update in the pathogenesis and treatment of chemotherapy and cancer induced anemia. Crit. Rev. Oncol. Hematol..

[25] Thomas M.R., Scully M. (2021). How I treat microangiopathic hemolytic anemia in patients with cancer. Blood.

[26] Leger R.M., Jain S., Nester T.A., Kaplan H. (2015). Drug-induced immune hemolytic anemia associated with anti-carboplatin and the first example of anti-paclitaxel. Transfusion.

[27] Spiegel M. (2024). Unveiling the Antioxidative Potential of Galangin: Complete and Detailed Mechanistic Insights through Density Functional Theory Studies. J. Org. Chem..

[28] Viskupicova J., Blaskovic D., Galiniak S., Soszyński M., Bartosz G., Horakova L., Sadowska-Bartosz I. (2015). Effect of high glucose concentrations on human erythrocytes in vitro. Redox Biol..

[29] Repsold L., Joubert A.M. (2018). Eryptosis: An Erythrocyte’s Suicidal Type of Cell Death. Biomed Res. Int..

[30] Bhavsar S.K., Gu S., Bobbala D., Lang F. (2011). Janus kinase 3 is expressed in erythrocytes, phosphorylated upon energy depletion and involved in the regulation of suicidal erythrocyte death. Cell. Physiol. Biochem..

[31] Calderón-Salinas J.V., Muñoz-Reyes E.G., Guerrero-Romero J.F., Rodríguez-Morán M., Bracho-Riquelme R.L., Carrera-Gracia M.A., Quintanar-Escorza M.A. (2011). Eryptosis and oxidative damage in type 2 diabetic mellitus patients with chronic kidney disease. Mol. Cell. Biochem..

[32] Niemoeller O.M., Bentzen P.J., Lang E., Lang F. (2007). Adenosine protects against suicidal erythrocyte death. Pflugers Arch..

[33] Semeraro F., Ammollo C.T., Esmon N.L., Esmon C.T. (2014). Histones induce phosphatidylserine exposure and a procoagulant phenotype in human red blood cells. J. Thromb. Haemost..

[34] Deng S., Hou G., Xue Z., Zhang L., Zhou Y., Liu C., Liu Y., Li Z. (2015). Vitamin E isomer δ-tocopherol enhances the efficiency of neural stem cell differentiation via L-type calcium channel. Neurosci. Lett..

[35] Zylinska L., Lisek M., Guo F., Boczek T. (2023). Vitamin C Modes of Action in Calcium-Involved Signaling in the Brain. Antioxidants.

[36] Minich D.M., Henning M., Darley C., Fahoum M., Schuler C.B., Frame J. (2022). Is Melatonin the “Next Vitamin D”?: A Review of Emerging Science, Clinical Uses, Safety, and Dietary Supplements. Nutrients.

[37] Yang B., Li S., Zhu J., Huang S., Zhang A., Jia Z., Ding G., Zhang Y. (2020). miR-214 Protects Against Uric Acid-Induced Endothelial Cell Apoptosis. Front. Med..

[38] Verzola D., Ratto E., Villaggio B., Parodi E.L., Pontremoli R., Garibotto G., Viazzi F. (2014). Uric acid promotes apoptosis in human proximal tubule cells by oxidative stress and the activation of NADPH oxidase NOX 4. PLoS ONE.

[39] Wang M., Wu J., Jiao H., Oluwabiyi C., Li H., Zhao J., Zhou Y., Wang X., Lin H. (2022). Enterocyte synthesizes and secrets uric acid as antioxidant to protect against oxidative stress via the involvement of Nrf pathway. Free Radic. Biol. Med..

[40] Roumeliotis S., Roumeliotis A., Dounousi E., Eleftheriadis T., Liakopoulos V. (2019). Dietary Antioxidant Supplements and Uric Acid in Chronic Kidney Disease: A Review. Nutrients.

[41] Dong Y., Han F., Su Y., Sun B., Zhao W., Pan C. (2023). High uric acid aggravates apoptosis of lung epithelial cells induced by cigarette smoke extract through downregulating PRDX2 in chronic obstructive pulmonary disease. Int. Immunopharmacol..

[42] Al Mamun Bhuyan A., Ashiqul Haque A.K.M., Sahu I., Cao H., Kormann M.S.D., Lang F. (2017). Inhibition of Suicidal Erythrocyte Death by Volasertib. Cell. Physiol. Biochem..

[43] Jemaà M., Fezai M., Lang F. (2017). Inhibition of Suicidal Erythrocyte Death by Reversine. Cell. Physiol. Biochem..

[44] Tkachenko A., Onishchenko A. (2023). Casein kinase 1α mediates eryptosis: A review. Apoptosis.

[45] Klarl B.A., Lang P.A., Kempe D.S., Niemoeller O.M., Akel A., Sobiesiak M., Eisele K., Podolski M., Huber S.M., Wieder T. (2006). Protein kinase C mediates erythrocyte “programmed cell death” following glucose depletion. Am. J. Physiol. Cell Physiol..

[46] Hazegh K., Fang F., Kelly K., Sinchar D., Wang L., Zuchelkowski B.E., Ufelle A.C., Esparza O., Davizon-Castillo P., Page G.P. (2022). Erythrocyte mitogen-activated protein kinases mediate hemolytic events under osmotic and oxidative stress and in hemolytic diseases. Cell. Signal..

[47] Gatidis S., Zelenak C., Fajol A., Lang E., Jilani K., Michael D., Qadri S.M., Lang F. (2011). p38 MAPK activation and function following osmotic shock of erythrocytes. Cell. Physiol. Biochem..

[48] Alfhili M.A., Alsughayyir J. (2024). Bufalin reprograms erythrocyte lifespan through p38 MAPK and Rac1 GTPase. Toxicon.

[49] Restivo I., Attanzio A., Giardina I.C., Di Gaudio F., Tesoriere L., Allegra M. (2022). Cigarette Smoke Extract Induces p38 MAPK-Initiated, Fas-Mediated Eryptosis. Int. J. Mol. Sci..

[50] Alzoubi K., Alktifan B., Oswald G., Fezai M., Abed M., Lang F. (2014). Breakdown of phosphatidylserine asymmetry following treatment of erythrocytes with lumefantrine. Toxins.

[51] Attanzio A., Frazzitta A., Cilla A., Livrea M.A., Tesoriere L., Allegra M. (2019). 7-Keto-Cholesterol and Cholestan-3beta, 5alpha, 6beta-Triol Induce Eryptosis through Distinct Pathways Leading to NADPH Oxidase and Nitric Oxide Synthase Activation. Cell. Physiol. Biochem..

[52] Lang E., Bissinger R., Gulbins E., Lang F. (2015). Ceramide in the regulation of eryptosis, the suicidal erythrocyte death. Apoptosis.

[53] Li D., Zheng X., Zhang Y., Li X., Chen X., Yin Y., Hu J., Li J., Guo M., Wang X. (2022). What Should Be Responsible for Eryptosis in Chronic Kidney Disease?. Kidney Blood Press. Res..

[54] Lang K.S., Myssina S., Lang P.A., Tanneur V., Kempe D.S., Mack A.F., Huber S.M., Wieder T., Lang F., Duranton C. (2004). Inhibition of erythrocyte phosphatidylserine exposure by urea and Cl^−^. Am. J. Physiol. Renal Physiol..

[55] Virzì G.M., Mattiotti M., Clementi A., Milan Manani S., Battaglia G.G., Ronco C., Zanella M. (2022). In Vitro Induction of Eryptosis by Uremic Toxins and Inflammation Mediators in Healthy Red Blood Cells. J. Clin. Med..

[56] Sparrow R.L., Sran A., Healey G., Veale M.F., Norris P.J. (2014). In vitro measures of membrane changes reveal differences between red blood cells stored in saline-adenine-glucose-mannitol and AS-1 additive solutions: A paired study. Transfusion.

[57] Alfhili M.A., Lee M.H. (2021). Flow Cytofluorometric Analysis of Molecular Mechanisms of Premature Red Blood Cell Death. Methods Mol. Biol..

[58] Jemaà M., Fezai M., Bissinger R., Lang F. (2017). Methods Employed in Cytofluorometric Assessment of Eryptosis, the Suicidal Erythrocyte Death. Cell. Physiol. Biochem..

[59] Zhbanov A., Yang S. (2015). Effects of Aggregation on Blood Sedimentation and Conductivity. PLoS ONE.

[60] Evans B.C., Nelson C.E., Yu S.S., Beavers K.R., Kim A.J., Li H., Nelson H.M., Giorgio T.D., Duvall C.L. (2013). Ex vivo red blood cell hemolysis assay for the evaluation of pH-responsive endosomolytic agents for cytosolic delivery of biomacromolecular drugs. J. Vis. Exp..

