# Supplementary figures and images for: Galangin Triggers Eryptosis and Hemolysis Through Ca2+ Nucleation and Metabolic Collapse Mediated by PKC/CK1α/COX/p38/Rac1 Signaling Axis
Source: Int J Mol Sci. 2024 Nov 15;25(22):12267. doi: 10.3390/ijms252212267 (PMC11594942; doi:10.3390/ijms252212267)

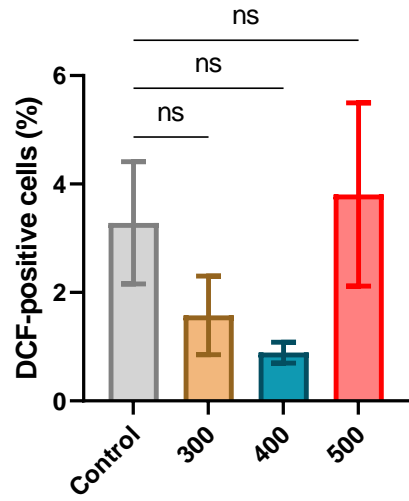

**Supplementary Figure S1: Lack of oxidative stress in GAL-induced RBC death.**

Supplement: Supplementary file 1 [file ijms-25-12267-s001.zip › ijms-3313202-supplementary.pdf]
